# Inactivation of group 2 σ factors upregulates production of transcription and translation machineries in the cyanobacterium *Synechocystis* sp. PCC 6803

**DOI:** 10.1038/s41598-018-28736-9

**Published:** 2018-07-09

**Authors:** Satu Koskinen, Kaisa Hakkila, Juha Kurkela, Esa Tyystjärvi, Taina Tyystjärvi

**Affiliations:** Department of Biochemistry, University of Turku, FI-20014 Turku, Finland

## Abstract

We show that the formation of the RNAP holoenzyme with the primary σ factor SigA increases in the Δ*sigBCDE* strain of the cyanobacterium *Synechocystis* sp. PCC 6803 lacking all group 2 σ factors. The high RNAP-SigA holoenzyme content directly induces transcription of a particular set of housekeeping genes, including ones encoding transcription and translation machineries. In accordance with upregulated transcripts, Δ*sigBCDE* contain more RNAPs and ribosomal subunits than the control strain. Extra RNAPs are fully active, and the RNA content of Δ*sigBCDE* cells is almost tripled compared to that in the control strain. Although Δ*sigBCDE* cells produce extra rRNAs and ribosomal proteins, functional extra ribosomes are not formed, and translation activity and protein content remained similar in Δ*sigBCDE* as in the control strain. The arrangement of the RNA polymerase core genes together with the ribosomal protein genes might play a role in the co-regulation of transcription and translation machineries. Sequence logos were constructed to compare promoters of those housekeeping genes that directly react to the RNAP-SigA holoenzyme content and those ones that do not. Cyanobacterial strains with engineered transcription and translation machineries might provide solutions for construction of highly efficient production platforms for biotechnical applications in the future.

## Introduction

Cyanobacteria are eubacteria characterized by oxygenic photosynthesis. They branched early from other eubacteria and are currently found where ever light is available, from oceans to hot springs and fresh water environments to desert crust. These important primary producers are estimated to be responsible for one-third of carbon fixation on Earth^1^. Recently cyanobacterial research has focused on possibilities to use cyanobacteria as bio-factories to produce valuable compounds. Lack of efficient, easily-controllable promoters has been a bottleneck in the application of cyanobacteria in biotechnology^2^. Although many *E*. *coli* promoters function in cyanobacteria and cyanobacterial promoters in *E*. *coli*, none of the commonly used *E*. *coli* production systems functions well in cyanobacteria. These experiences suggest that transcriptional regulation differs notably between cyanobacteria and *E*. *coli*.

The RNA polymerase (RNAP) has gained specific features in the cyanobacterial lineage. In the vast majority of eubacteria, the RNAP core is composed of two α subunits and a single β, β′ and ω subunit. In the cyanobacterial lineage, the RNAP core has a unique composition with six subunits. The β′ subunit has been split in cyanobacteria, and the N-terminal part is called γ and the C-terminal part retains the name β′^3^. Only the plastid encoded RNAPs of plants and algae share this arrangement^4^. As plastids are descendants of cyanobacteria, the splitting of β′ has obviously occurred only once in evolution. The biological reasons and consequences of β′ splitting remain to be solved. The β′ subunit bears a 600 amino acids long cyanobacteria lineage specific insertion^5^ whose physiological role remains unclear as well. The small ω subunit of RNAP is non-essential in cyanobacteria just like in many other eubacteria^6^, and it might play a regulatory role in cyanobacteria^7^.

For transcription initiation, the RNAP core recruits one of the several σ factors to form a transcription initiation competent RNAP holoenzyme. All σ factors compete for the same RNAP core^8^. This competition is affected by numerous factors including the amount of each σ factor, affinity of different σ factors to the RNAP core, amounts and activities of anti-σ factors that prevent recruitment of a particular σ factor, and RNAP modifying factors like the small signaling molecule ppGpp that directly binds to the RNAP core and changes the recruitment efficiency of different σ factors and affect the promoter selectivity of the RNAP holoenzyme^8–10^. These processes are not yet well understood in cyanobacteria.

Cyanobacteria typically encode multiple group 2 σ factors that closely resemble the essential primary σ factor but are non-essential in standard growth conditions^11–13^. The primary σ factor is assumed to be mainly responsible for transcription of housekeeping genes during growth. In a particular stress condition, the recruitment of one or more of the group 2 σ factors increases. Accordingly cells lacking group 2 σ factors show acclimation defects in high salt^11,14,15^, bright light^12,16,17^ or heat stresses^18–20^. In addition to stress responses, the group 2 σ factors control metabolic changes during dark-light transitions^17,21–24^.

We have constructed a *Synechocystis* sp. PCC 6803 strain without any functional group 2 σ factors^13^. The Δ*sigBCDE* strain is vulnerable to all tested stress conditions but grows well in standard conditions^13^. However, even in the standard conditions almost 20% of genes are at least two-fold up or down regulated in Δ*sigBCDE* compared to the control strain^13^. Interestingly, many genes encoding subunits of the transcriptional or translational machineries are upregulated in the Δ*sigBCDE* mutant. In the present paper, we further investigated the regulation of transcriptional and translational machineries in cyanobacteria. The results indicate that in the absence of competition between SigA and group 2 σ factors, more RNAP-SigA holoenzyme is formed than in the control strain. Numerous RNAP-SigA holoenzymes enhance the transcription of a particular set of housekeeping genes including those encoding transcription and translation machineries, and Δ*sigBCDE* produces more RNAPs and ribosomal subunits than the control strain. The extra RNAPs are fully functional, and enhanced transcription leads to a high RNA content per cell whereas extra ribosomal subunits do not form translationally active ribosomes, and Δ*sigBCDE* cells show similar translation activity and protein content as the control strain. Co-regulation of transcription and translation machineries as well as promoter differences between subgroups of housekeeping genes will be discussed.

## Results

### Transcripts for transcription and translation machineries are abundant in the Δ*sigBCDE* strain

We have previously shown that the Δ*sigBCDE* strain that lacks all functional group 2 σ factors grows well in standard growth conditions^13^. DNA microarray analysis of the transcriptome of Δ*sigBCDE* in the standard growth conditions revealed that many genes encoding proteins for transcription and translation machineries were up-regulated in the Δ*sigBCDE* strain^13^ (Supplementary Table S1). These comprise genes for RNAP core subunits, tRNA synthases, ribosomal protein subunits and translation initiation, elongation and termination factors. Unlike the RNAP core genes, the *sigA* gene, encoding the primary σ factor in *Synechocystis*, was down-regulated (Supplementary Table S1). The opposite behavior of genes encoding RNAP core and *sigA* promoted us to study the amount and activity of RNAP complexes in the mutant strain.

### RNAP content and transcription activity are upregulated in Δ*sigBCDE*

**Figure 1:**
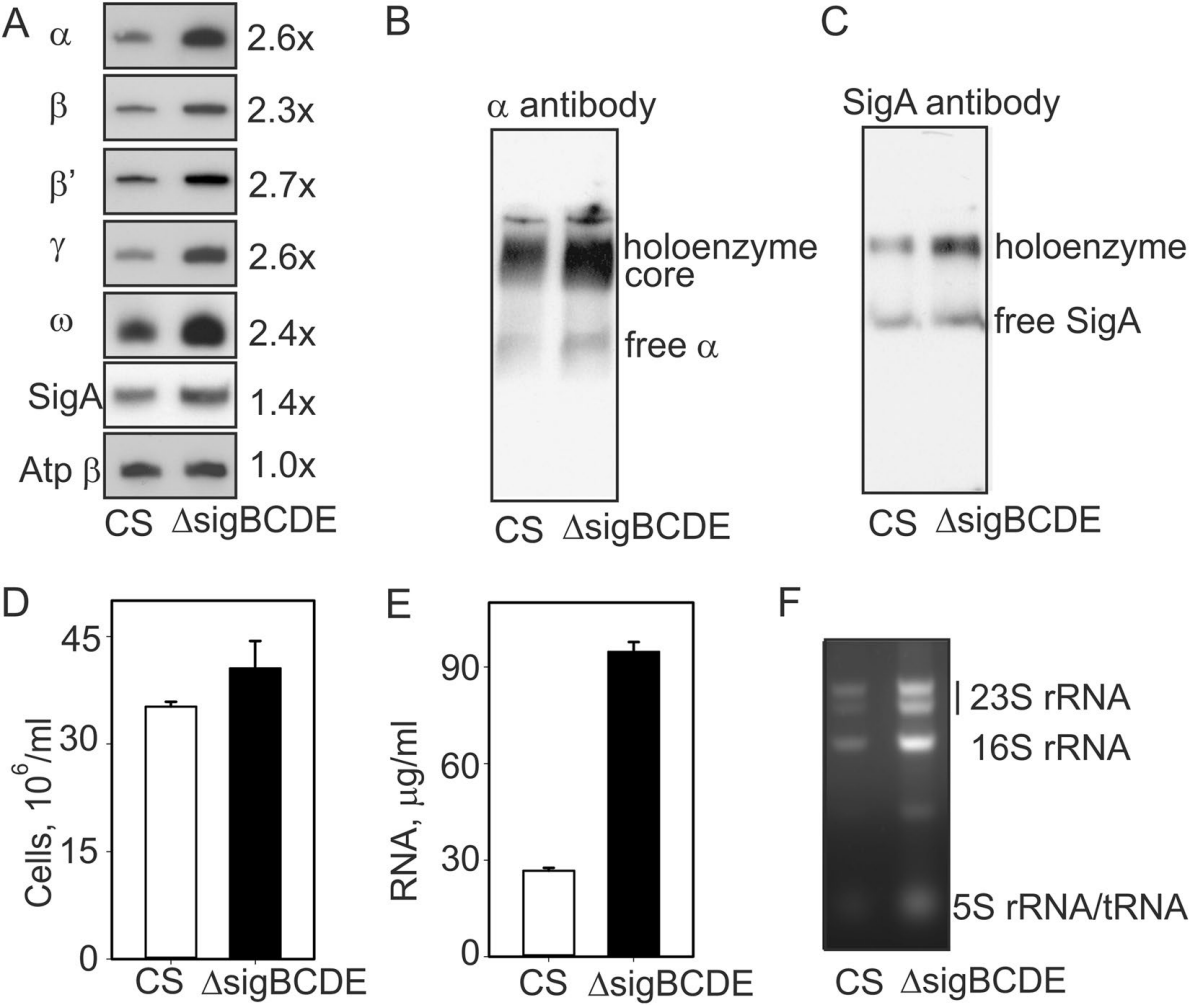
The RNA polymerase and RNA contents of the Δ*sigBCDE* and control (CS) strains. (**A**) Cells were grown is standard conditions, total proteins were isolated and separated by SDS-PAGE, and RNA polymerase core proteins and the primary σ factor SigA were detected by western blotting. As a loading control the amount of the β subunit of ATPase was detected as well. Original Western blots are shown in Supplementary Fig. S1. (**B**,**C**) Cells were grown is standard growth conditions, proteins were isolated and samples containing 60 μg of soluble proteins were separated by blue native gel electrophoresis, transferred to the membrane and the α subunit of the RNA polymerase core (**B**) and the primary σ factor SigA (**C**) were detected with specific antibodies. (**D**) The number of cells in mL of cell culture with OD_730_ of 1. Three independent cell cultures were analyzed, bars denote SD. Student’s t-test P = 0.175. (**E**) Total RNAs were isolated from the same amount of the cells and the RNA concentration was detected. Six independent samples were analyzed. Student’s t-test P = 4.5 × 10^−9^ F) A 5 μL-sample of isolated RNA was separated by 1.2% agarose gel electrophoresis and stained with ethidium bromide.

To measure the amount of the RNAP in Δ*sigBCDE*, total proteins were isolated from cells grown in standard conditions and the amounts of RNAP subunits were detected by western blotting. The RNAP core proteins α, β, β′, γ and ω were approximately 2.5-fold more abundant in Δ*sigBCDE* than in the control strain (CS) (Fig. 1A; original Western blots are shown in Supplementary Fig. S1). Similar amount of the ATP synthase β subunit in both strains confirmed equal loading of the samples in western blots. Thus increased transcripts of RNAP core genes were indeed used to produce more RNAPs. In addition, the SigA protein was 1.4-fold more abundant in Δ*sigBCDE* than in CS (Fig. 1A) although *sigA* transcripts were 6.1-fold down-regulated in Δ*sigBCDE* (Supplementary Table S1) indicating either post-transcriptional regulation of the *sigA* gene or higher stability the SigA protein in the mutant than in CS.

To analyze RNAP complexes, isolated protein samples were treated with ServaG blue and separated by blue native gel electrophoresis. ServaG blue binds to protein complexes and proteins, giving a negative charge without disturbing the subunit composition of the complex. After electrophoresis, proteins were transferred to a membrane and then the SigA factor and the α subunit were detected by western blotting. The antibody against the α subunit detected a wide area and a faint band (Fig. 1B). The upper part of the wide area contains the RNAP holoenzyme complex with the σ factor, as this part is also recognized by the SigA antibody (Fig. 1C). The lower part of the wide area contains the RNAP core complex without the σ factor (Fig. 1B,C). The faint band contains unassembled α subunits (Fig. 1C). The RNAP-SigA holoenzyme was more abundant in Δ*sigBCDE* than in the control strain while the amount of free SigA protein did not differ between the strains (Fig. 1C). Also the RNAP core complexes were more abundant in Δ*sigBCDE* than in CS (Fig. 1B).

The RNAP holoenzyme with the primary σ factor is assumed to transcribe housekeeping genes during active growth. Upregulation of RNAP-SigA holoenzyme in Δ*sigBCDE* might thus enhance transcription. To figure out if this is the case, the RNA content of cells of Δ*sigBCDE* and CS was analyzed. Cell counting with a flow cytometer confirmed that the cell number per OD_730_ was similar in both strains (Fig. 1D). The total amount of RNA isolated from 1 ml of cell culture with optical density at 730 nm of 1.0 was approximately three-fold as high in Δ*sigBCDE* as in CS (Fig. 1E) showing that the extra RNAPs of Δ*sigBCDE* are active. The vast majority of all cellular RNAs are ribosomal RNAs, and analysis of RNA by agarose gel electrophoresis revealed a high rRNA content in Δ*sigBCDE*. Ribosomal RNA genes form two identical operons in *Synechocystis*^25^ and the final rRNAs are maturated in a complex process including cleavages of the sugar-phosphate backbone and modification of the bases. Only mature rRNAs were detected (Fig. 1F) indicating that not only transcription of rRNA operons was upregulated in the mutant strains but these extra RNAs were efficiently processed to mature rRNAs.

### Upregulation of ribosomal subunits does not enhance translation in Δ*sigBCDE*

**Figure 2:**
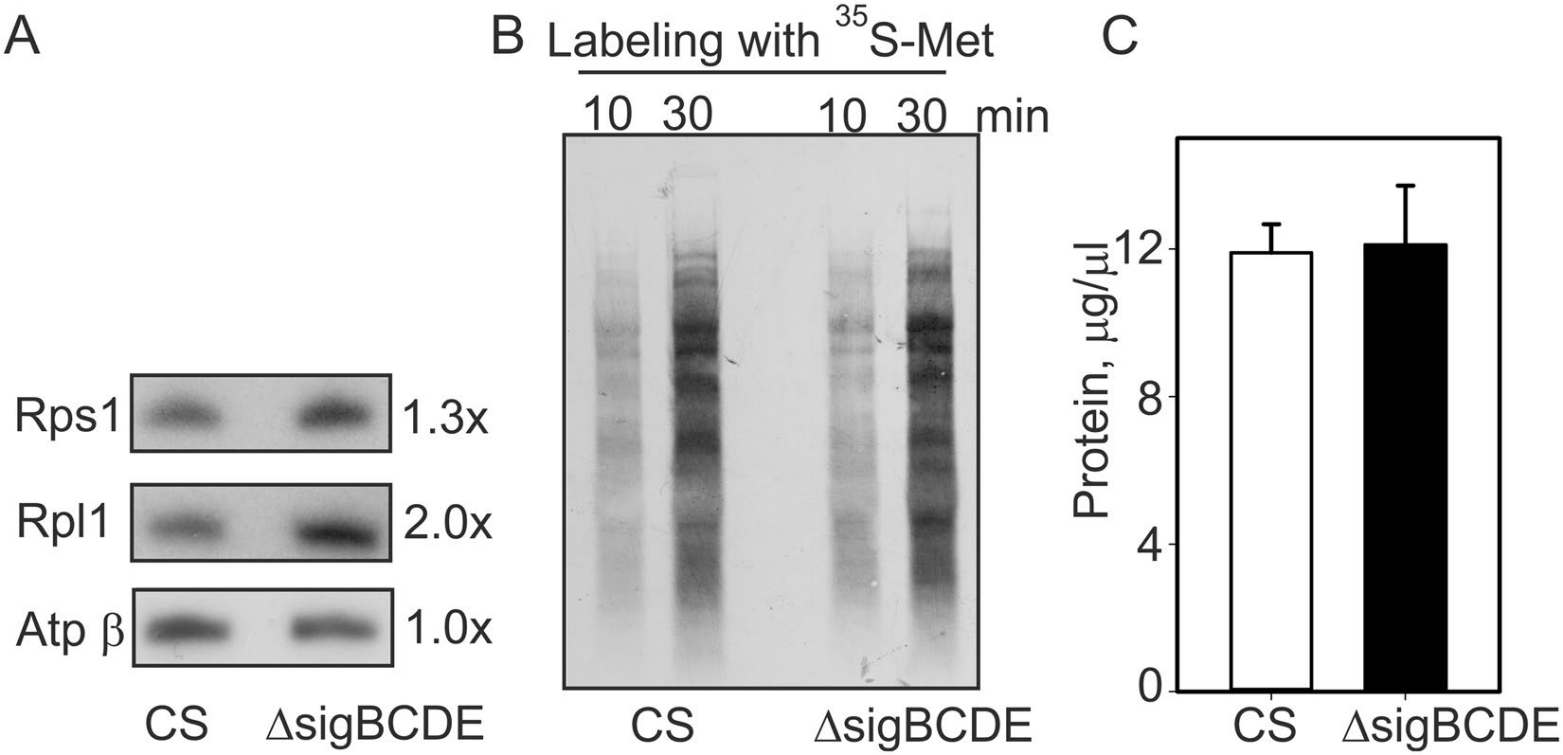
Ribosomal proteins, translation activity and total protein contents in the Δ*sigBCDE* and control (CS) strains in standard growth conditions. (**A**) Total proteins were isolated, separated by SDS-PAGE and then the Rps1 protein of the 30S ribosomal subunit and the Rpl1 protein of the 50S ribosomal subunit were detected with western blotting. As a loading control the amount of the β subunit of ATPase was detected as well. Original Western blots are shown in Supplementary Fig. S1. (**B**) Cells were labelled with radioactive ^35^S-methionine for 10 or 30 min, as indicated, in standard growth conditions, and total proteins were isolated after addition of excess of cold methionine. Proteins were separated by SDS-PAGE, transferred to the membrane and visualized by autoradiography. (**C**) Total proteins were isolated from the same amount of cells and the protein content was detected with a BioRad DC protein assay kit. Three independent biological replicates were measured, Student’s t-test P = 0.905.

The synthesis rate of ribosomal proteins in *E*. *coli* depends on the rRNA content of the cells. We found that also in Δ*sigBCDE* the high rRNA content was accompanied with a high amount of the Rps1 (subunit of 30S ribosomal particle) and the Rpl1 (subunit of 50S ribosomal particle) proteins (Fig. 2A: original Western blots are shown in Supplementary Fig. S1). However, labelling newly synthesized protein with radioactive ^35^S-methionine revealed that translation activity was similar in both strains (Fig. 2B) as was also the protein content of cells (Fig. 2C). Thus, an increased amount of ribosomal subunits did not accelerate translation in Δ*sigBCDE*.

**Figure 3:**
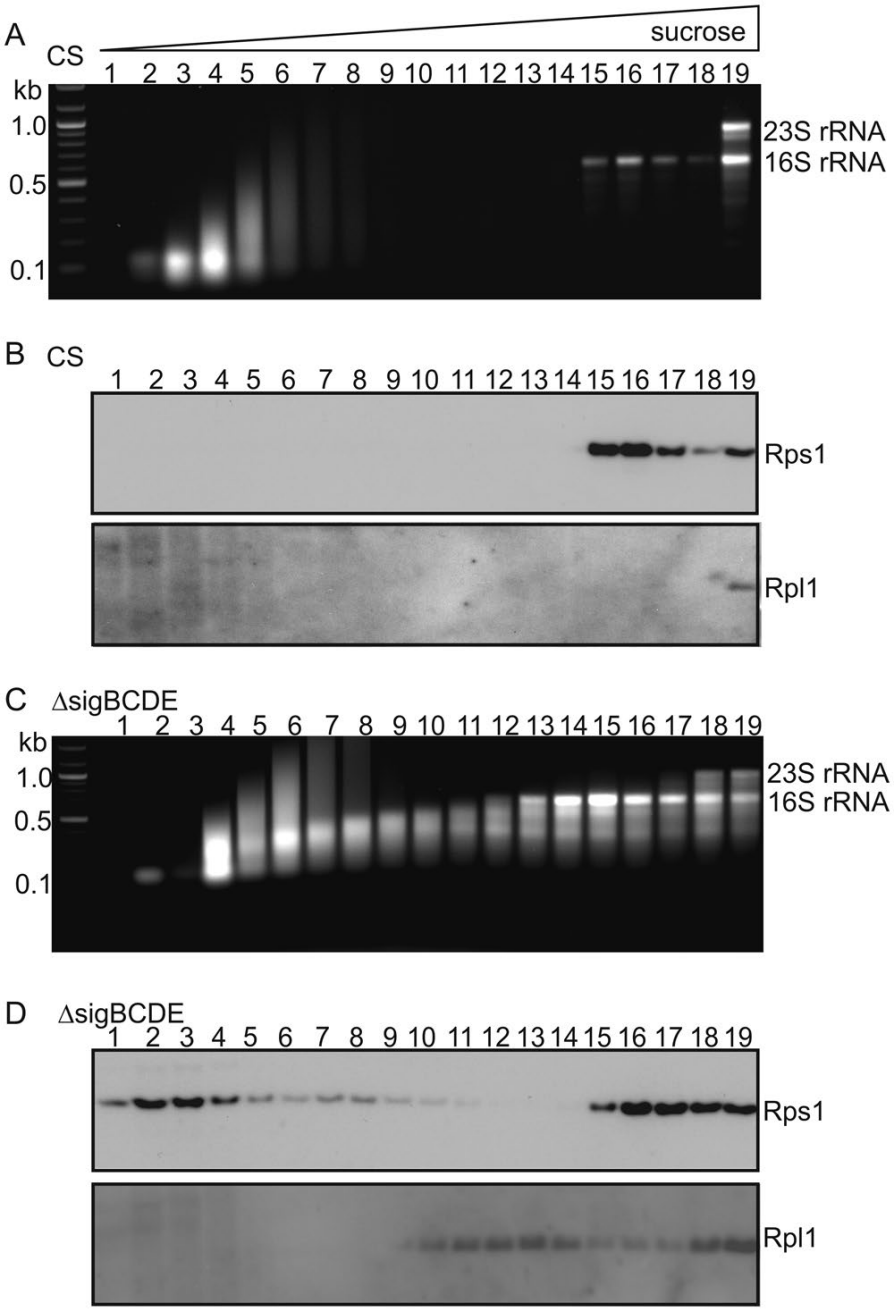
Ribosome profiles of the control (CS) and Δ*sigBCDE* strains. The ribosomes of the CS (**A**,**B**) and Δ*sigBCDE* (**C**,**D**) strains were fractionated with a 5–35% sucrose density gradient centrifugation, nineteen fractions were collected and either RNA or ribosomal proteins Rps1 and Rpl1 were detected from each fraction. RNA was isolated from the 300 μL sample of fraction, dissolved in 20 μL of water, and 15 μL of isolated RNA from each fraction of CS (**A**) or Δ*sigBCDE* (**C**) was loaded on a 1.2% agarose gel and stained with ethidium bromide. The DNA molecular marker was included to demonstrate separation of fragments. For western blots, a 24 μL sample of a fraction was solubilized and proteins were separated on SDS-PAGE and Rps1 and Rpl1 proteins were detected by specific antibodies by western blotting in the control (**B**) and ΔsigBCDE (**D**) strains.

To find reason(s) for the discrepancy between the ribosomal subunit content and translation activity we further analyzed the ribosomes of Δ*sigBCDE*. To that end, a soluble protein fraction containing ribosomes was isolated and separated by sucrose density gradient centrifugation. To keep 70S ribosomes intact, buffers were supplemented with 10 mM Mg^2+^. After the centrifugation, nineteen fractions were collected, and RNAs and the Rps1 and Rpl1 proteins were analyzed from each fraction. In CS, all ribosomal subunits (16S and 23S rRNAs and the Rps1 and Rpl1 proteins) were only detected in Fraction 19 (Fig. 3A,B) whereas in Δ*sigBCDE* all these ribosomal subunits were detected in Fractions 18 and 19 (Fig. 3C,D). Although the overall number of translating ribosomes (Fractions 18–19) was similar in both strains, the results indicate that polysomes (Fraction 19) are more common in CS than in Δ*sigBCDE* while in Δ*sigBCDE* monosomes (Fraction 18) are also typical. As the amount of translating ribosomes was similar in both strains but many housekeeping genes produce more copies of mRNA in Δ*sigBCDE* than in CS, the number of ribosomes reading each mRNA molecule must be lower in Δ*sigBCDE* than in CS.

Fractions 15–17 contained 16S rRNA and the Rps1 protein in both strains indicating that these fractions represent 30S ribosomal particles (Fig. 3). In addition, the Rps1 protein was detected in Fractions 1–11 in Δ*sigBCDE* but not in CS. As Fractions 1–11 did not contain full length 16S rRNA, they do not represent functional ribosomal particles. In the lightest fractions, Rps1 might be a free protein while in the other fractions it might form complexes with some other ribosomal proteins and partially degraded 16S rRNA, seen as shorter RNA species. Similarly, the Rpl1 proteins and shorter RNA fragments in Fractions 10–17 detected in the Δ*sigBCDE* strain might represent functionally incompetent 50S ribosomal particles (Fig. 3C,D).

During the assembly of 30S and 50S ribosomal particles, protein subunits are attached to the 16S and 23S rRNAs, respectively. Light complexes containing partially degraded rRNAs and part of ribosomal protein subunits are common in Δ*sigBCDE* but not in CS (Fig. 3). As all ribosomal proteins were not upregulated at the transcriptional level in Δ*sigBCDE* (Supplementary Table S1), it is possible that the assembly of extra ribosomal particles is never fully completed before the partially assembled ribosomes are degraded in Δ*sigBCDE*.

### Arrangement of RNAP core genes in *Synechocystis* sp. PCC 6803 genome

Our results indicate that the production of the main components of transcriptional and translational machineries were upregulated Δ*sigBCDE*. Interestingly, the gene encoding the α subunit of the RNAP core is surrounded by numerous genes encoding ribosomal proteins and thus arrangement of these genes into an operon could provide a basis for co-regulation of transcriptional and translational machineries. To detect if *rpoA* forms an operon with surrounding genes, DNA-free RNA was isolated and cDNA synthesis was performed using random hexanucleotide primers. Specific primer pairs recognizing two adjacent genes were then used in subsequent PCR reactions. Successful amplification of the DNA fragment in a PCR reaction indicates that the adjacent genes are in the same cDNA molecule thus belonging to the same operon.

**Figure 4:**
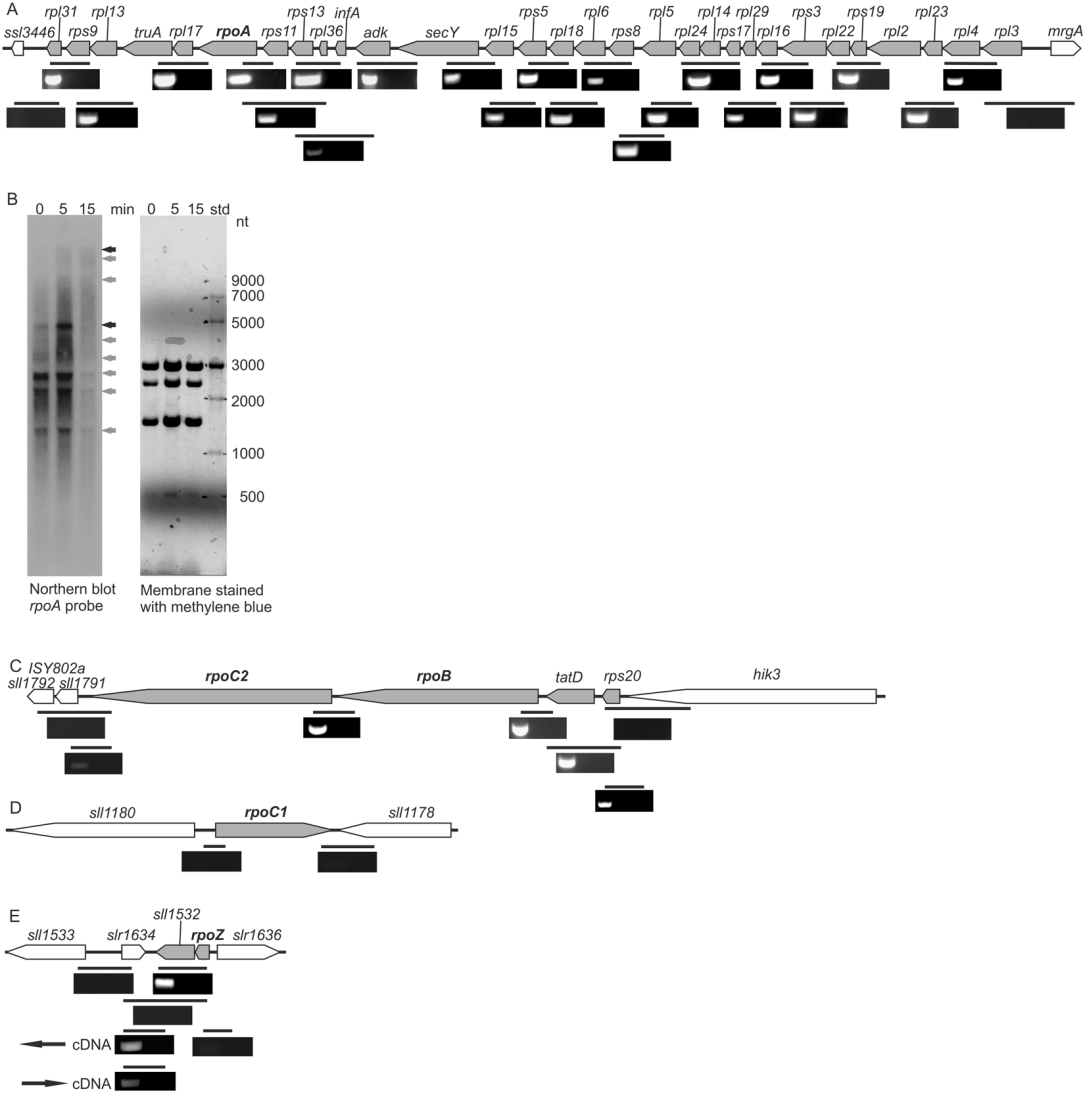
Arrangement of genes encoding the RNAP core proteins in the genome of *Synechocystis* sp. PCC 6803. To test which genes are transcribed in the same unit, DNA-free RNA was isolated from *Synechocystis* and used as a template for cDNA synthesis using random hexamer primers and reverse transcriptase. Specific primers were thereafter used to amplify the indicated regions in the genome by PCR, and the PCR products were separated with 0.8% agarose gels and stained with ethidium bromide. The left lane is always a reaction with reverse transcriptase and the right lane is a control reaction without the reverse transcriptase. (**A**) The *rpoA* operon. (**B**) The Northern blot analysis using the *rpoA* probe. The cells were treated with transcription initiation inhibitor, rifampicin, for 0, 5 or 15 min at the standard growth conditions, the RNAs were isolated with hot phenol method and separated with glyoxal-phosphate buffer system using 1.2% agarose gel and transferred to the membrane. The *rpoA* gene was amplified from the genomic DNA of *Synechocystis* with PCR, label with ^32^P-dCTP and the Northern hybridization was performed with the standard procedure. The black arrows indicate approximately 16000 and 4700 nt long transcripts and the other main transcripts are pointed with grey arrows. (**C**) The *rpoC2*-*rpoB* operon. (**D**) The *rpoC1* gene. (**E**) The *rpoZ* operon. Arrows indicate the direction of the cDNA synthesis when specific primers were used in cDNA synthesis instead of random hexamers. Original agarose gels for (**A**,**C**–**E**) are shown in Supplementary Fig. S2.

The *rpoA* gene was found to belong to a large gene complex where 30 genes showed positive signals for an operon test (Fig. 4A; original agarose gels are shown in Supplementary Fig. S2). In addition to the *rpoA* gene, this region comprises 8 genes for 30S ribosomal proteins, 17 genes for 50S ribosomal proteins, the *infA* gene encoding the translation initiation factor IF-1, the *secY* gene encoding a subunit of the protein translocase, the *adk* gene encoding adenylate kinase and the *truA* gene encoding the tRNA pseudouridine synthase (Fig. 4A). However, when expression levels of these 30 genes were compared, two genes, *adk* and *infA*, in the middle of the region, showed lower expression than the other genes (Supplementary Table S2). In an earlier transcriptome analysis, this region was divided to four transcription units *rpl3*-*rpl15* (TU837), *secY*-*adK* (TU836), *infA* (Tu835), *rpl36*-*rpl31* (TU833)^26^. Our RNA secondary structure analysis revealed possible transcription termination loops downstream of the *rpl15* and *secY* genes. Because combining all these results turns out to be complicated, we next performed a Northern blot analysis.

Cells were treated with a transcription initiation inhibitor, rifampicin for 0, 5 or 15 min and transcripts were detected using the *rpoA* probe. The longest transcripts were circa 16 000 nt and could thus comprise genes from *rpl3* to *rpl31* (Fig. 4B). However, these long transcripts were not abundant. Instead, an approximately 4700 nt long transcript (this size would fit to the predicted TU833 from *rpl36* to *rpl31*) were abundant together with 2700 nt, 2200 nt and 1500 nt long transcripts (Fig. 4B). The longest transcripts became more abundant when transcription initiation was prevented with rifampicin. Thus, actively transcribing cells might mainly produce the numerous short transcripts form *rpl3*-*rpl31* while the full length *rpl3*-*rpl31* transcripts are mainly produced only if transcription initiation inside the operon is not active. The longest transcript was more stable than the shorter ones (Fig. 4B). Our results indicate a complicated transcription pattern for the *rpoA* region including many internal transcription initiation and termination sites, possible regulation via antitermination process resulting in partially overlapping transcripts covering the whole region from *rpl3* to *rpl31*.

The *rpoC2* (the β′ subunit) and *rpoB* (the β subunit) genes form an operon together with the *tatD* gene (subunit of the twin-arginine protein export system) and the *rps20* gene (a subunit of 30S ribosome), while the *rpoC1* gene (the γ subunit) produces a monocistronic transcript (Fig. 4C,D). The *rpoZ* gene forms an operon with the *sll1532* gene with an unknown function (Fig. 4E). Transcription of both the *slr1634* gene and the *rpoZ*-*sll1532* operon continues long downstream the actual coding regions so that a great part of this area is transcribed to both directions. The originally sequenced glucose tolerant Kazusa strain of *Synechocystis* sp. PCC 6803 contains a probable transposase gene *slr1635* between the *rpoZ* and *slr1636* genes, but *slr1635* is missing from our CS and from many other sequenced *Synechocystis* strains^6^.

### Promoter regions of differently regulated housekeeping genes

**Figure 5:**
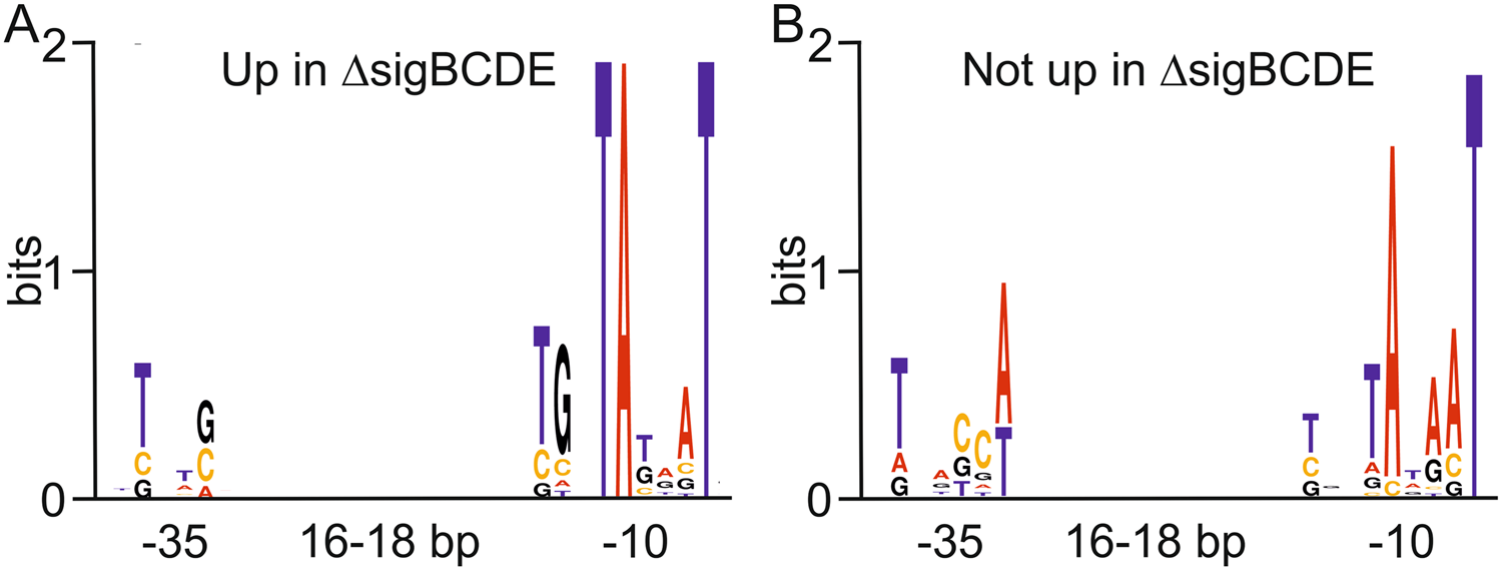
Sequence logos of promoter regions of the selected highly expressed genes in ΔsigBCDE. (**A**) Genes upregulated in Δ*sigBCDE*. (**B**) Genes showing similar or lower expression in Δ*sigBCDE* than in the control strain.

Our results revealed that housekeeping genes in *Synechocystis* can be divided to two categories, to those that are upregulated when RNAP-SigA content increases and to those that do not directly respond to RNAP-SigA content. We analyzed *in silico* the promoter regions of those operons/genes that were upregulated in Δ*sigBCDE* and those that were not affected or were even down-regulated in Δ*sigBCDE*. Only genes/operons with experimentally verified transcription initiation sites were chosen^26,27^. The expression levels of the selected genes in Δ*sigBCDE*, in comparison to CS, are indicated in Supplementary Table S3. An extended −10 region characterized by TGN just upstream of the −10 region was detected in many genes upregulated in Δ*sigBCDE* but not in those ones showing similar or lower expression in Δ*sigBCDE* than in CS (Fig. 5) suggesting that an extended −10 region type promoter can be considered as a consensus promoter for genes that respond directly to an increased amount of the RNAP-SigA holoenzyme. Genes that were not up-regulated in Δ*sigBCDE* showed high variability in the −35 region and shared only the conserved −10 region with classical *E*. *coli* promoters (Fig. 5).

## Discussion

### Regulation of the primary σ factor

The regulation of the formation of the RNAP-SigA holoenzyme is a complex process in *Synechocystis*. Transcriptional down-regulation of *sigA* in *Synechocystis* has been shown in many stress conditions like high temperature, high salt^21^ or nitrogen deficiency^28^. Furthermore, the *sigA* transcripts diminish in the night and reappear again after the onset of illumination^21^. The group 2 σ factor SigB is required for rapid activation of *sigA* transcription after onset of light^21^ suggesting that at least the RNAP-SigB holoenzyme, in addition to the RNAP-SigA holoenzyme, can recognize *sigA* prometer(s). In standard growth conditions the RNAP-SigB holoenzyme content is low^13^ and thus the regulation of the *sigA* gene by the SigB factor is important only in special conditions like dark-light transitions. The *sigA* gene is located just upstream of the *rrn* operon^25^ but not co-transcribed with the *rrn* operon^21^. Currently three different promoters have been identified for the sigA gene. The *sigA* gene has suggested to form a transcription unit with a non-coding RNA *ncr1650*^27^. A typical −10 region is found upstream of *ncr1650* but the −35 region is unique (Supplementary Fig. S3). Imamura and co-workers^29^ identified two other promoters closer to the initiation codon (Supplementary Fig. S3). In addition to the transcriptional regulation, low content of the *sigA* mRNA in Δ*sigBCDE* but high abundance of the SigA protein indicates regulation at post-transcriptional level. The mechanism of this regulation remains to be solved, but one possibility is that extra SigA protein triggers down-regulation of *sigA* transcripts.

In Δ*sigBCDE*, a high amount of SigA leads to extra efficient formation of the RNAP-SigA holoenzyme. However, the recruitment of SigA by the RNAP core does not depend only on the abundance of SigA. The *Synechocystis* strain missing the non-essential ω subunit of the RNAP core recruits less SigA than CS although the SigA protein is available^6^. During nitrogen starvation, the RNAP-SigA holoenzyme content decreases more prominently than the SigA protein content and simultaneously the recruitment of group 2 σ factors increases^28^. In *E*. *coli*, unfavorable conditions induce production of the small signaling molecule ppGpp that binds to the RNAP core and renders the core less favorable to recruitment of σ^70^ and more favorable to the group 2 σ factor σ^S^ ^9^. In addition, stationary phase induces production of the Rsd protein that functions as an anti-σ^70^ factor in *E*. *coli*, thereby decreasing the formation of the RNAP-σ^70^ holoenzyme^30^. Furthermore, binding of the RNAP-σ^70^ holoenzyme to the small 6S RNA molecule prevents active transcription in *E*. *coli* until favorable conditions are resumed (for 6S RNA regulation, see review^31^). All together, these regulatory mechanisms lower the transcription activity of housekeeping genes in *E*. *coli* a lot without large changes in the σ^70^ content. Unlike in *E*. *coli*, the SigA content of cells drops drastically in the stationary phase in *Synechocystis*, whereas 6S RNA content increases only slightly^28^, suggesting that the dose of the primary σ factor might play a more direct role in controlling the formation of the RNAP holoenzyme in *Synechocystis* than in *E*. *coli*.

### RNAP-SigA holoenzyme and growth rate

In eubacteria, all σ factors compete for the same RNAP core to form a transcription initiation competent RNAP-holoenzyme, different σ factors having different affinity to the core^8^. In Δ*sigBCDE*, an elevated dose of SigA factor and a low amount of competitors result in formation of a high number of RNAP-SigA holoenzymes, over expression of many housekeeping genes and accumulation of extra RNAs. However, the growth rate of the Δ*sigBCDE* is not elevated^13^, indicating that overall transcriptional enhancement does not induce faster growth and on the other hand that accumulation of extra RNA is not harmful for *Synechocystis* cells. However, when the growth rate of *Synechocystis* changes due to changed environmental conditions, the growth rate and formation of RNAP-SigA holoenzymes often correlate. For example, in high CO_2_ the formation of RNAP-SigA holoenzymes is abundant and cells grow fast^7^ whereas nitrogen depletion causes cessation of growth and minimal formation of RNAP-SigA holoenzymes^28,32^. *E*. *coli* cells produce different amounts of RNAP depending on growth medium, and the RNAP level has been shown to be just above the level needed for maximal growth in the particular growth medium^33^. In *E*. *coli*, similarly as in *Synechocystis*, simultaneous overproduction of RNAP core and the primary σ^70^ factor enhances transcription of many housekeeping genes but does not enhance growth^34^.

### Selection of promoters by RNAP-SigA holoenzyme

In the Δ*sigBCDE* strain, a high content of RNAP-SigA holoenzyme induces expression of particular housekeeping genes^13^. We selected a collection of upregulated and non-upregulated housekeeping genes in Δ*sigBCDE* and compared their promoters. Upregulated *Synechocystis* promoters often resembled *E*. *coli* promoters with an extended −10 region^35^. The promoters of non-upregulated genes only contain a typical −10 region while the rest of the promoter region varies from gene to gene. Thus promoters recognized by RNAP-SigA holoenzyme in *Synechocystis* contain a clear −10 region but otherwise those promoters show high variability, suggesting that housekeeping genes are regulated in a complicated manner that requires other factors in addition to the RNAP holoenzyme. Increased availability of RNAP-SigA holoenzymes directly enhanced the transcription efficiency of genes with extended −10 region promoters in *Synechocystis*, while cyanobacterial-specific promoters of the photosynthetic machinery did not respond to the overdose of RNAP-SigA holoenzyme. Thus, our results suggest that activation of photosynthesis related genes requires more specific regulators than just a high content of RNAP-SigA holoenzyme. Demand for mechanisms strictly regulating expression of photosynthetic genes is obvious, as imbalances in the function of photosynthetic reactions potentially induces production of harmful reactive oxygen species in cyanobacteria^36^. We have previously shown that in the Δ*rpoZ* strain, a low RNAP-SigA holoenzyme content lowers especially the transcription of genes encoding the carbon concentrating and carbon fixating machineries while expression of many other housekeeping genes remain at the normal level^6^. Patterns of gene expression in the Δ*sigBCDE* and Δ*rpoZ* strains show that different functional groups of housekeeping genes respond differently to the dose of the RNAP-SigA holoenzyme. This might be a common phenomenon in bacteria, as a recent study showed that the dose sensitivity of the RNAP-σ^S^ holoenzyme correlates with the functional category of the σ^S^ regulated genes in *E*. *coli*^37^.

### Regulation of translation machinery

The Δ*sigBCDE* strain produces more rRNAs than CS. In *E*. *coli* activity of rRNA operons correlates with growth rate^38^ and the presence of thirteen highly active rRNA operons in *Vibrio natriegens* has been use to explain the rapid growth of this bacterium (doubling time of only 10 min)^39^. High rRNA content but normal growth of Δ*sigBCDE* shows that growth rate and rRNA content do not directly correlate in *Synechocystis*.

Two major regulatory signals, the concentration of initiator trinucleotides (ATP for six operons and GTP for one) and the amount of the starvation induced ppGpp molecule, set the expression level of the rRNA operons in *E*. *coli*^40^. The *rrn* operons of *E*. *coli* contain two promoters P1 and P2; P2 provides rather constant expression while P1 is highly induced or repressed depending on conditions^38^. The high transcription efficiency from the P1 promoters has been explained by their unique features that lead to formation of a scrunched open complex, thereby reducing abortive initiation^41^. The two identical *rrn* operons of *Synechocystis* have GTP as an initiator nucleotide and they contain only a single promoter resembling the σ^70^ consensus promoter with neither an extended −10 region nor a discriminator sequence typical for the P1 promoter (Supplementary Fig. S4), suggesting that cyanobacterial and *E*. *coli rrn* operons are regulated differently. Furthermore, a Blast search revealed that *Synechocystis* lacks close homologs of the HN-S, DksA or Fis proteins regulating rRNA operons in *E*. *coli*^38,40,42^. In *Mycobacterium tuberculosis* the CarD protein replaces the function of the DksA protein^43^ but close homologs of this protein are missing in *Synechocystis* as well.

*Synechocystis* does contain Nus proteins that regulate Rho-dependent attenuation of the *rrn* operon in *E*. *coli*^38^, but the Rho factor is missing and thus an *E*. *coli* type attenuation mechanism cannot function in cyanobacteria. Nus proteins in *Synechocystis* might be involved in rRNA processing in the same way as was recently reported in *E*. *coli*^44^. At least rRNAs are efficiently processed in Δ*sigBCDE*, as only 16S, 23S and 5S rRNAs were detected and according to microarray data^13^ transcripts of many Nus proteins and rRNA processing enzymes were up-regulated in Δ*sigBCDE*.

Ribosomal RNA content regulates the production of ribosomal proteins with a feedback mechanism in which ribosomal proteins bind strongly to rRNA and weakly to their own mRNA^45^. In accordance with that, the high rRNA content of Δ*sigBCDE* is accompanied with high ribosomal protein content in the mutant strain. Further studies are required to reveal the actual mechanism regulating ribosome production in *Synechocystis*.

### Co-regulation of transcription and translation machineries

Our results show that the production of both transcription and translation machineries are similarly up-regulated in the Δ*sigBCDE* strain. Interestingly, both the *rpoA* operon and the *rpoB*-*rpoC2* operon contain ribosomal protein genes in addition to RNAP genes. Actually the long *rpoA* cluster in *Synechocystis* comprises three ribosomal protein operons of *E*. *coli* (Supplementary Fig. S5). Gene order near the *rpoA* gene seems to be conserved. Analysis of circa 5000 bacterial genomes reveals that in eubacteria, the arrangement of *rps13*, *rps11*, *and rpl17* genes next to the *rpoA* gene is highly conserved, and in many cases also *rps4* is part of the gene cluster (Supplemental Table S5). In *Synechocystis*, the *rps4* gene forms a monocistronic transcript in a separate location, and the *rpoA* operon encodes numerous other ribosomal genes in addition to the *rps13*, *rps11*, *rpl17* genes. High conservation of *rps13*-*rps11*-(*rps4*)-*rpoA*-*rpl17* gene cluster suggests that the *rpoA* operon might play a central role for simultaneous production of transcriptional and translational machineries. The biogenesis of the RNAP core starts with the formation of the α dimer^46^ but the actual roles of Rps13, Rps11 and Rpl17 proteins in the formation of functional ribosomes during the complicated assembly of ribosomes (for a review see^47^) remains to be elucidated.

## Conclusions

**Figure 6:**
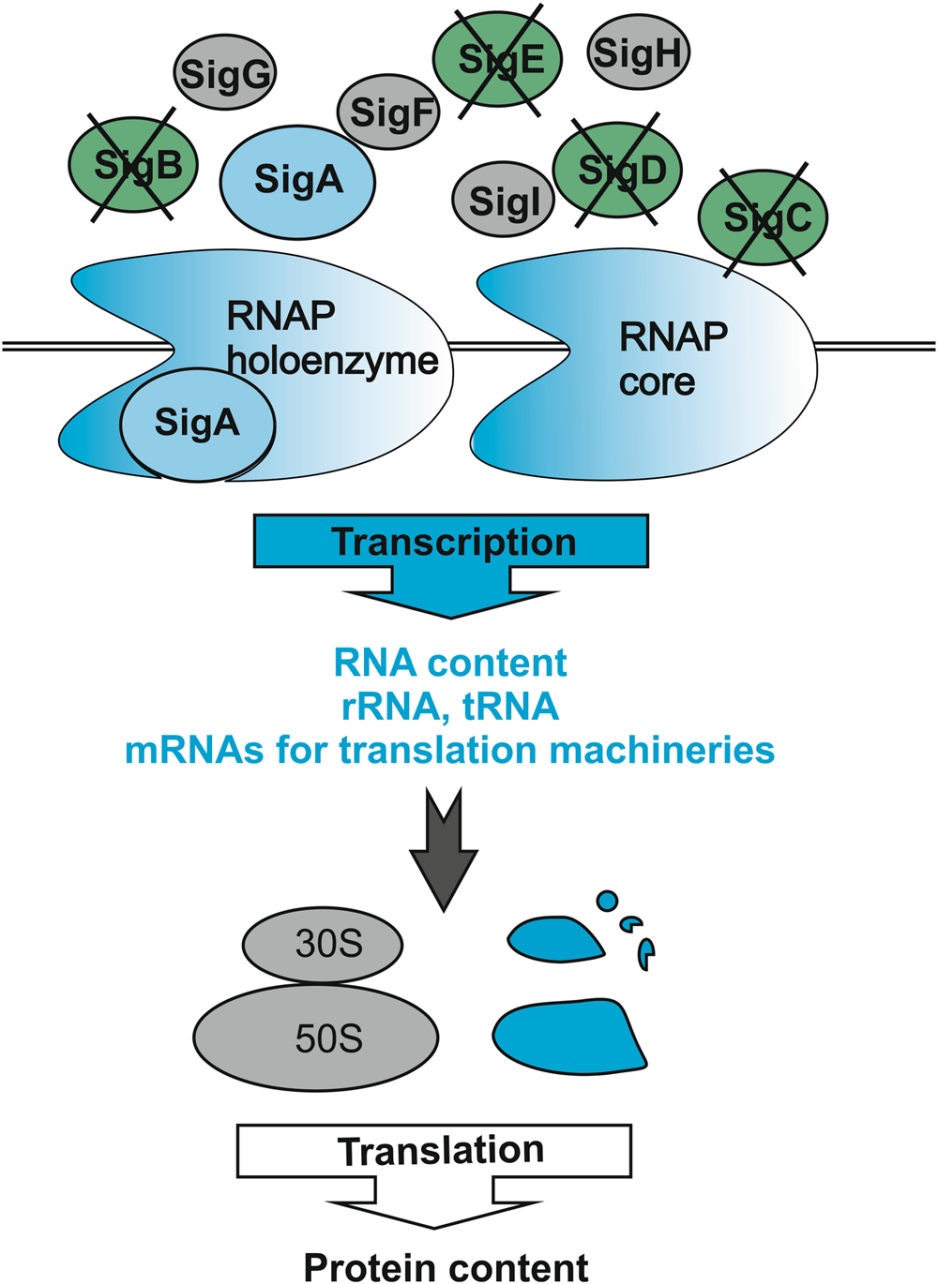
A model of the regulation of the gene expression machinery in *Synechocystis*. Components or processes up-regulated in Δ*sigBCDE* are shown in blue. The formation of RNAP-SigA holoenzyme is enhanced in Δ*sigBCDE* because there is no competition with group 2 σ factors. High RNAP-SigA content leads to higher transcription activity of particular housekeeping genes including transcription and translation machinery genes. The extra transcripts are used to produce extra RNAPs that are transcriptionally active and produce up-normally high RNA content in Δ*sigBCDE* cells including rRNAs and tRNAs that are part of the translation machinery. In addition to rRNA, also ribosomal subunits are produced in excess in Δ*sigBCDE* but extra ribosomal subunits do not form functional ribosomes, the translation activity in Δ*sigBCDE* remains similar as in the control strain and also protein content in Δ*sigBCDE* cells is similar as in the control strain.

Our findings about cyanobacterial regulation of transcriptional and translational machineries are summarized in Fig. 6. Inactivation of group 2 σ factors increases the content of the principal σ factor SigA and the formation of the RNAP-SigA holoenzyme, which leads to an increase in the transcription of many housekeeping genes including genes for RNAP itself and the translational machinery. Extra transcripts of the RNAP subunits are used to produce high amounts of transcriptionally active RNAP, which enhances transcription and triples the RNA content of the cell. Ribosomal subunits are also produced in excess in Δ*sigBCDE*, but translation activity and the total protein content of the control and Δ*sigBCDE* strains are similar. The amount of functional ribosomes in Δ*sigBCDE* remains similar as in the control strain, pointing to post-translational regulation of the number of active ribosomes. A possibility to engineer a cyanobacterial strain with high transcription and translation capacity without a high growth rate might offer new solutions for the use of cyanobacteria for efficient production of valuable compounds in future.

## Materials and Methods

### Strains and growth conditions

The glucose-tolerant strain of *Synechocystis* sp. PCC 6803 (CS) and Δ*sigBCDE* were grown in BG-11 medium buffered with 20 mM Hepes, pH 7.5, in normal air at 32 °C under constant illumination at the photosynthetic photon flux density of 40 µmol m^−2^ s^−1^ as described earlier^13^. The BG-11 agar plates of Δ*sigBCDE* were supplemented with appropriate antibiotics but for the experiments, liquid cultures were grown without antibiotics.

### Cell content of the culture

The cell content was detected by measuring OD_730_, dense cultures were diluted to OD_730_ = 0.4 before measurements. To determine the cell number of the CS and Δ*sigBCDE* cultures, the OD_730_ was set to 0.6 and cells were calculated with a flow cytometer BD LSRRortessa (BD Biosciences).

### Analyses of RNA polymerase complexes

To analyze the amounts of the RNAP subunits, 30 mL of culture (OD_730_ was 1.0) grown in standard conditions was harvested at 4 °C by centrifugation at 4500 g for 6 min. Then total proteins were isolated as described earlier^12^ and protein concentration was measured with Bio-Rad DC protein assay kit. Then 15 µg of proteins were solubilized and separated with 10% NEXT GEL^®^ SDS-PAGE (Amresco), transferred to Immobilon-P membrane (Millipore) with Trans-Blot^®^ (BioRad) according to the manufacturer’s instructions. The RNAP core proteins and the primary σ factor SigA were detected by Western blotting as described^13^. Immunoblots were quantified using a FluorChem image analyzer (Alpha Innotech Corp). As a loading control, ATPase β subunit was detected with a specific antibody (Agrisera). Three independent biological replicates were analyzed.

The RNAP complexes were analyzed by blue native gel electrophoresis. Cells were grown and harvested as describe above. The cell pellet was washed with BN isolation buffer (50 mM TrisHCl pH 7.5, 150 mM NaCl, 10 mM NaEDTA pH 8.0, supplemented with Protease Inhibitor Cocktail (Roche)). Then 180 µL of BN isolation buffer and the pellet volume of acid-washed 150–212 µm glass beads (Sigma) were added, and cells were broken by vortexing 8 × 1 min at 4 °C. Cell debris and membranes were removed by two centrifugation steps, first at 10 000 g for 5 min and then 18 000 g for 15 min.

To separate protein complexes, 60 μg of soluble proteins were solubilized with BN solubilization buffer (Serva G Blue 5 mg mL^−1^, 2% glycerol, 90 mM sucrose, 2.5 mM BisTris) for 5 min on ice, and separated using 5–12% gradient gels (acrylamide:bis-acrylamide 32:1) with 4% of stacking gel using Mini protean gel system (BioRad) at 4 °C. The anode buffer was 50 mM BisTris pH 7.0 and the cathode buffer 50 mM Tricine, 15 mM BisTris pH 7.0 supplemented with 0.01% Serva G; the voltage was increased after 30 min of run from 75 V to 100 V and again after 30 min to 150 V. Then after 30 min, the cathode buffer was changed to a similar buffer without Serva G and the run was continued at 200 V until the blue color ran out of the gel. The gel was soaked in a transfer buffer (48 mM Tris, 39 mM glycine, 0.0375% SDS and 20% methanol) for 30 min and thereafter protein transfer and immunodetection was performed as described above. Two independent biological replicates were analyzed.

### RNA content of cells

Total RNAs was isolated from 1-ml cell culture (OD_730_ was 1) with the hot phenol method as described earlier^48^, and any DNA contamination was removed with TURBO DNA-free^TM^ kit (Ambion). The RNA content was measured with a spectrophotometer at 260 nm (Biodrop). To visualize RNA, a sample of isolated RNA from the same amount of the cells was separated on 1.2% agarose gel and stained with ethidium bromide. The DNA microarray data^13^ is available in GEO accession GSE69981.

### Operon analysis

DNA-free RNA was extracted from CS as described earlier^6^. Reverse transcription (RT) was performed for 900 ng RNA using SuperScript III kit (Invitrogen) according to the manufacturer’s instructions. Then 2 µL of RT reaction was used in sequent PCR amplification with Phusion HeatShock II (Thermo Scientific). The PCR primers are listed in Supplemental Table S5. To detect PCR products, 5 µL of the PCR products were separated on 1% agarose gel and stained with ethidium bromide.

### Northern blot analysis

The cell culture (30 mL; OD_730_ was 1) was supplemented with rifampicin (500 μg mL^−1^) and cells were collected by centrifugation after 0, 5 or 15 min of incubation in the standard growth conditions. RNA was isolated with the hot-phenol method^48^. Samples containing 8 μg of total RNA were denatured with the glyoxal system, separated on 1.2% agarose gel in phosphate buffer and transferred to a Hybond-N membrane^48^. The *rpoA* gene was amplified from genomic DNA using specific primers (Supplementary Table S5) and labelled with α-^32^P-dCTP 10 mCi mL^−1^ (Perkin Elmer) with the Prime-a-gen labelling system (Promega) according to the manufacturer’s instructions. After prehybridization in 6xSSC, 1xDenhardt’s, 0.1% SDS, 100 μg mL^−1^ Herring sperm DNAS for 1 h at 67 °C, denatured probe was added and hybridized overnight at 67 °C. Then membrane was washed once with 2xSCC, 0.1% SDS at 60 °C for 15 min and once with 1xSCC, 0.1% SDS at 60 °C for 15 min and autoradiographed.

### Promoter analysis

Two groups of promoters of highly expressed genes were selected for sequence analysis. The first group comprises genes up-regulated in Δ*sigBCDE* and the second group consists of genes that either show similar expression in Δ*sigBCDE* and CS or are down-regulated in Δ*sigBCDE* (Supplementary Table S3). Each of the selected genes contained a well-defined transcriptional start site, obtained from previous studies^26,27^ and the sequence 1–60 nt upstream of each start site was retrieved. For modeling of the −10 region, the sequences were aligned, maximizing the information content in a 6-nt window ending 8 nt upstream of the transcription start site. In the optimization of the alignment, each sequence was allowed to move by 1 nt to the left and to the right. The −35 motif was modeled by aligning the sequences initially according to the −10 region, and then maximizing the information content of a 6-nt window located 15–17 nt upstream of the −10 region. The alignments were optimized with custom software used earlier in^6^.

### Ribosome analysis

Cells (100 mL, OD_730_ was 1) were collected from standard conditions by centrifugation and ribosomes were isolated at 4 °C. The cell pellet was rapidly washed with 20 mM Na-EDTA, 20 mM Tris-HCl pH 8 and then with isolation buffer (20 mM NH_4_Cl, 10 mM MgCl_2_, 5 mM dithiothreitol, 20 mM Tris-HCl pH 8.0, and protease inhibitor cocktail (Roche)). The pellet was resuspended in 2 mL of isolation buffer and the cells were broken using a French press (Constant Systems Ltd) treating the samples twice at 2.7 kPa. Cell debris was removed by centrifugation at 10 000 g for 10 min, and the supernatant was centrifuged again at 30 000 g for 30 min. The supernatant was collected and protein concentration was determined. Two mg of proteins were loaded onto the top of 5% to 45% linear sucrose density gradient in isolation buffer and the samples were centrifuged in TH-641 rotor (Sorval) at 107000 g for 18 h at 4 °C. After centrifugation, 19 fractions, 620 μL of each, were collected. Three independent biological replicates were analyzed.

Ribosomal proteins were analyzed by western blotting. A 24-μL sample of each fraction was solubilized and proteins were separated on 12% SDS-PAGE gels with 4% stacking gel (acrylamide: bis-acrylamide 37.5:1) using Protean^®^ II (BioRad). Proteins were transfer to the membrane and immunodetected as described above. The Rps1 (AS08309) and Rpl1 (AS111738) antibodies were purchased from Agrisera.

Total RNAs were isolated from each fraction. An aliquot of 300 μL of a fraction was mixed with 200 μL of RNA isolation buffer (20 mM Na-acetate pH 4.5, 2% SDS, 250 mM Na-EDTA) and treated with one unit of DNaseI (Life Technologies) for 5 min and then extracted once with phenol:chlororoform (1:1) and once with chloroform. RNAs were precipitated at −20 °C overnight after addition of LiCl to a final concentration of 0.725 M and 2.5 volumes of ethanol. RNAs were collected by centrifugation at 18 000 g for 15 min and the RNA pellet was washed once with 70% ethanol and dissolved into 20 μL of water. Fifteen μL samples were separated on 1.2% agarose gels in TAE buffer and stained with ethidium bromide.

### Detection of newly synthesized proteins

Cells were grown in standard growth conditions. Five μL of radioactive ^35^S-methionine (35S L-Met 185 MBq, Perkin Elmer) was added to 20 mL of cell culture (OD_730_ was 1) and cells were labelled for 10 or 30 min under the standard growth conditions. Cold L-Met was added to a final concentration 0.4 mg mL^−1^, samples were rapidly cooled and cells were harvested by centrifugation at 4500 g for 6 min at 4 °C and total proteins were isolated as described^15^. Proteins (60 μg) were separated by 10% NEXT GEL SDS-PAGE, transferred onto membrane and autoradiographed. Two independent biological replicates were performed.

## Electronic supplementary material

Supplementary information

Dataset 1
