# Supplementary material for: Inactivation of group 2 σ factors upregulates production of transcription and translation machineries in the cyanobacterium Synechocystis sp. PCC 6803
Source: Sci Rep. 2018 Jul 9;8:10305. doi: 10.1038/s41598-018-28736-9 (PMC6037674; doi:10.1038/s41598-018-28736-9)
Supplement: Supplementary file 1 — Supplementary information [file 41598_2018_28736_MOESM1_ESM.pdf]

Supplementary material

Inactivation of group 2 σ factors upregulates production of transcription and translation machineries in the cyanobacterium *Synechocystis* sp. PCC 6803

Satu Koskinen, Kaisa Hakkila, Juha Kurkela, Esa Tyystjärvi and Taina Tyystjärvi*

Department of Biochemistry, University of Turku, FI-20014 Turku, Finland

Table S1. Comparison of transcript levels of selected transcription and translation related genes in Δ*sigBCDE* and control strains of *Synechocystis* sp. PCC 6803 in standard growth conditions. **Pages 2-4**
Table S2. Transcript abundance and predicted transcription units for genes next to the RNA polymerase core genes in *Synechocystis* sp. PCC 6803. **Page 5**
Table S3. Promoters of highly expressed genes being either up-regulated or showing similar or lower expression in Δ*sigBCDE* than in the control strain were selected for promoter analysis. P**age 6**
Table S4. The organization of the *rps13, rps11, rps4, rpoA* and *rpl17* genes in 5080 bacterial genomes **(A separate Exel Table).**
Table S5. Primers used in this study and the length of the PCR products. **Pages 7-8**
Figure S1. Original western blots for Figures 1 and 2. **Page 9**
Figure S2. Original agarose gels for operon analysis. **Page 10**
Figure S3. The upstream region of the sigA gene. **Page 11**
Figure S4. The identical promoter regions of the two *rrn* operons of *Synechocystis* sp. PCC 6803. **Page 11**
Figure S5. Comparison of the *rpoA* operons in *Synechocystis* and in *E.coli*. **Page 11**

Table S1. Comparison of transcript levels of selected transcription and translation related genes in ΔsigBCDE and control strains of Synechocystis sp. PCC 6803 in standard growth conditions. Fold changes are presented as log_2 of the ratio ΔsigBCDE/CS. Original data are available in GEO (accession GSE69981).

Code	Gene symbol	log_2 FC	p-value	Protein
sll1818	rpoA	0.97	0.029	RNA polymerase alpha subunit
sll1787	rpoB	0.49	0.029	RNA polymerase beta subunit
sll1789	rpoC2	0.72	0.014	RNA polymerase beta prime subunit
slr1265	rpoC1	0.62	0.001	RNA polymerase gamma-subunit
ssl2982	rpoZ	-0.51	0.007	RNA polymerase omega subunit
slr0653	sigA	-2.61	0.001	SigA
slr1564	sigF	-0.54	0.001	SigF
sll0856	sigH	0.92	0.029	SigH
sll0687	sigI	-0.17	0.374	SigI
slr1545	sigG	0.03	0.896	SigG
slr0743	nusA	0.69	0.040	similar to N utilization substance protein
sll0271	nusB	-0.44	0.008	N utilization substance protein B homolog
ssl3441	infA	1.53	0.005	initiation factor IF-1
slr0744	infB	-0.60	0.052	translation initiation factor IF-2
slr1938		1.00	0.005	putative EIF-2b subunit 1
slr0974	infC	-0.71	0.065	initiation factor IF-3
sll0546		0.27	0.085	probable translation initiation factor
sll1261	tsf	1.49	0.000	elongation factor TS
slr0434	efp	0.90	0.001	elongation factor P
sll1098	fus	1.07	0.005	elongation factor EF-G
sll0830	fus	-0.98	0.007	elongation factor EF-G
slr1463	fus	0.78	0.007	elongation factor EF-G
sll1099	tufA	0.05	0.378	elongation factor Tu
sll0145	rrf	1.36	0.002	ribosome releasing factor
sll1110	prfA	0.51	0.003	peptide chain release factor 1
sll1865	pfbB	0.71	0.001	peptide chain release factor 2
slr1228	prfC	-0.02	0.956	peptide-chain-release factor 3
sll1074	leuS	0.62	0.000	leucyl-tRNA synthetase
slr0958	cysS	1.18	0.000	cysteinyl-tRNA synthetase
slr0357	hisS1	0.74	0.003	histidyl-tRNA synthetase
slr1031	tyrS	1.05	0.005	tyrosyl tRNA synthetase
sll0362	alaS	0.52	0.008	alanyl-tRNA synthetase
sll0179	gltX	0.35	0.010	glutamyl-tRNA synthetase
sll1553	pheT	0.90	0.011	phenylalanyl-tRNA synthetase
slr1884	trpS	0.74	0.012	tryptophanyl-tRNA synthetase
sll0495	asnS	0.40	0.033	asparaginyl-tRNA synthetase
sll0078	thrS	0.37	0.060	threonyl-tRNA synthetase
slr1560	hisS2	0.14	0.104	histidyl tRNA synthetase

slr0557	valS	-0.19	0.171	valyl-tRNA synthetase
slr1703	serS	-0.17	0.326	seryl-tRNA synthetase
slr0649	metS	-0.29	0.394	methionyl-tRNA synthetase
sll1362	ileS	-0.06	0.455	isoleucyl-tRNA synthetase
sll0502	argS	0.06	0.647	arginyl-tRNA-synthetase
slr1550	lysS	0.05	0.747	lysyl-tRNA synthetase
slr1720	aspS	0.01	0.908	aspartyl-tRNA synthetase
sll0454	pheS	0.91	0.000	phenylalanyl-tRNA synthetase alpha chain
sll0927	metX	1.01	0.007	S-adenosylmethionine synthetase
slr0638	glyQ	0.76	0.000	glycyl-tRNA synthetase alpha chain
slr0220	glyS	0.46	0.058	glycyl-tRNA synthetase beta chain
slr1592		-0.70	0.006	probable pseudouridine synthase
sll1820	truA	0.64	0.050	tRNA pseudouridine synthase 1
slr0612		0.31	0.098	probable pseudouridine synthase
slr0457	truB	0.40	0.158	tRNA pseudouridine synthase B
sll1744	rpl1	1.33	0.006	50S ribosomal protein L1
sll1802	rpl2	0.86	0.021	50S ribosomal protein L2
sll1799	rpl3	1.27	0.021	50S ribosomal protein L3
sll1800	rpl4	0.99	0.026	50S ribosomal protein L4
sll1808	rpl5	1.00	0.005	50S ribosomal protein L5
sll1810	rpl6	1.05	0.001	50S ribosomal protein L6
sll1244	rpl9	0.25	0.038	50S ribosomal protein L9
sll1745	rpl10	1.00	0.002	50S ribosomal protein L10
sll1743	rpl11	1.10	0.009	50S ribosomal protein L11
sll1746	rpl12	1.02	0.002	50S ribosomal protein L12
sll1821	rpl13	0.34	0.152	50S ribosomal protein L13
sll1806	rpl14	1.02	0.009	50S ribosomal protein L14
sll1813	rpl15	1.26	0.004	50S ribosomal protein L15
sll1805	rpl16	0.44	0.094	50S ribosomal protein L16
sll1819	rpl17	0.90	0.027	50S ribosomal protein L17
sll1811	rpl18	0.94	0.002	50S ribosomal protein L18
sll1740	rpl19	0.25	0.108	50S ribosomal protein L19
sll0767	rpl20	-0.36	0.002	50S ribosomal protein L20
slr1678	rpl21	-0.14	0.228	50S ribosomal protein L21
sll1803	rpl22	0.80	0.030	50S ribosomal protein L22
sll1801	rpl23	0.96	0.024	50S ribosomal protein L23
sll1807	rpl24	0.81	0.015	50S ribosomal protein L24
sll1824	rpl25	0.79	0.004	50S ribosomal protein L25
ssr2799	rpl27	-0.12	0.165	50S ribosomal protein L27
ssr1604	rpl28	-0.01	0.941	50S ribosomal protein L28
ssl3436	rpl29	0.55	0.039	50S ribosomal protein L29
ssl3445	rpl31	0.76	0.022	50S ribosomal protein L31
ssr1736	rpl32	-0.53	0.031	50S ribosomal protein L32

ssr1398	rpl33	-0.04	0.689	50S ribosomal protein L33
smr0011	rpl34	0.61	0.010	50S ribosomal protein L34
ssl1426	rpl35	-0.32	0.004	50S ribosomal protein L35
sml0006	rpl36	0.63	0.051	50S ribosomal protein L36
slr1356	rps1	0.12	0.322	30S ribosomal protein S1
sll1260	rps2	0.80	0.002	30S ribosomal protein S2
sll1804	rps3	0.72	0.034	30S ribosomal protein S3
slr0469	rps4	0.86	0.000	30S ribosomal protein S4
sll1812	rps5	1.17	0.007	30S ribosomal protein S5
sll1767	rps6	0.50	0.043	30S ribosomal protein S6
sll1097	rps7	0.09	0.602	30S ribosomal protein S7
sll1809	rps8	1.05	0.004	30S ribosomal protein S8
sll1822	rps9	0.12	0.413	30S ribosomal protein S9
sll1101	rps10	0.63	0.013	30S ribosomal protein S10
sll1817	rps11	0.69	0.067	30S ribosomal protein S11
sll1096	rps12	-0.15	0.384	30S ribosomal protein S12
sll1816	rps13	0.71	0.047	30S ribosomal protein S13
slr0628	rps14	0.25	0.072	30S ribosomal protein S14
ssl1784	rps15	-0.06	0.823	30S ribosomal protein S15
ssr0482	rps16	-0.97	0.000	30S ribosomal protein S16
ssl3437	rps17	0.82	0.014	30S ribosomal protein S17
ssr1399	rps18	0.88	0.001	30S ribosomal protein S18
ssl3432	rps19	0.70	0.033	30S ribosomal protein S19
ssl2233	rps20	0.85	0.000	30S ribosomal protein S20
ssl0601	rps21	0.31	0.003	30S ribosomal protein S21
sll1814	secY	1.36	0.001	preprotein translocase SecY subunit
sll1815	adk	1.53	0.003	adenylate kinase
sll1786	tatD	0.90	0.002	putative deoxyribonuclease, tatD homolog

Table S2. Transcript abundance and predicted transcription units for genes next to the RNA polymerase core genes in *Synechocystis* sp. PCC 6803. The log_2 value of the signal intensity for each transcript in the standard conditions was taken from DNA microarray data (Gunnelius et al., 2014) and the transcript units are according to Mitschke et al. 2011.

Gene	Log_2 signal (1)	Transcription unit (2)
rpl3	14.70	TU837
rpl4	15.43	TU837
rpl23	15.20	TU837
rpl2	14.74	TU837
rps19	15.29	TU837
rpl22	15.19	TU837
rps3	14.97	TU837
rpl16	15.04	TU837
rpl29	14.48	TU837
rps17	14.53	TU837
rpl14	14.48	TU837
rpl24	14.81	TU837
rpl5	14.27	TU837
rps8	14.35	TU837
rpl6	14.45	TU837
rpl18	14.7	TU837
rps5	14.17	TU837
rpl15	14.38	TU837
secY	13.5	TU836
adK	12.01	TU836
infA	11.63	TU835
rpl36	14.07	TU833
rps13	14.18	TU833
rps11	14.35	TU833
rpoA	14.12	TU833
rpl17	13.87	TU833
truA	14.25	TU833
rpl13	16.61	TU833
rps9	14.84	TU833
rpl31	12.77	TU833
rps20	14.08	TU865
tatD	13.98	TU865
rpoB	13.64	TU865
rpoC2	13.14	TU865
rpoC1	13.28	TU1933
rpoZ	12.99	TU2128
sll1532	11.81	TU2128

Gunnelius,L., Hakkila,K., Kurkela,J., Wada,H., Tyystjärvi,E. and Tyystjärvi,T. (2014) The omega subunit of the RNA polymerase core directs transcription efficiency in cyanobacteria. *Nucleic Acids Res.*, **42,** 4606-4614.

Mitschke,J., Georg,J., Scholz,I., Sharma,C.M., Dienst,D., Bantscheff,J., Voss,B., Steglich,C., Wilde,A., Vogel,J., et al. (2011) An experimentally anchored map of transcriptional start sites in the model cyanobacterium *Synechocystis* sp. PCC6803. *Proc. Natl. Acad. Sci. U. S. A.*, **108,** 2124-2129.

Table S3. Promoters of selected genes. Highly expressed genes being either up-regulated or showing similar or lower expression in Δ*sigBCDE* than in the control strain were selected for promoter analysis. The expression ratio of these genes between the strains is indicated as Log_2 values of the fold change (FC). The data for protein coding genes is available in GEO (accession GSE69981) and data for tRNA (calculated as tRNA/cells) has been collected from our unpublished RNAseq analysis. The -10 and -35 elements and the length of the spacer between them are indicated.

Code	Gene or operon	-10	Spacer	-35	FC
Up-regulated in ΔsigBCDE					
sll1321	atp operon	tgaTATGAT	18	TAAGAA	1.26
sll1799	rpoA operon	ggcTATGAT	16	TGACGG	1.27
ssl2233	rpoC2-rpoB operon	tgtTAGAAT	16	TGTCGT	0.85
slr1265	rpoC1	tcgTACAAT	17	CATCCA	0.62
sll0927	metX	ccaTAGAAT	16	TTAACG	1.01
sll0691	pheS	tggTATTAT	16	GCTCTG	1.20
sll1553	pheT	cgaTACAAT	16	CCTGGG	0.90
slr0434	efp operon	tagTAGAAT	16	CTTCAA	0.90
sll1028	carboxysome operon	tccTATGCT	17	TGTCAA	1.28
slr0774	secD operon	cgaTAGAAT	16	TAGGGA	1.03
slr1510	plsX	tgcTACAAT	16	TCTGCT	1.01
slr0384	sqdx	cgaTATAGT	17	GGAGCA	1.00
6803t04	tRNA-Pro	tgtTAGGAT	16	TCTGAA	2.09
6803t09	tRNA-Trp	gggTATGAT	17	TGGGAA	1.48
6803t10	tRNA-Leu	tgaTATTAT	17	CTTAAT	0.88
6803t16	tRNA-Phe	tggTATGTT	16	TGTCAA	1.00
6803t17	tRNA-Gly	tgtTAGTCT	18	GACAAA	2.06
6803t18	tRNA-Ile	gttTAGAAT	18	TCTCCT	1.79
6803t19	tRNA-Arg	tgaTAGTGT	17	TGCGGA	1.01
6803t20	tRNA-Ser	cacTATTGT	18	CCAAAT	1.26
6803t21	tRNA-Ser	tgcTATGAT	16	GGCGGA	0.55
6803t22	tRNA-His	tgcTAGCAT	16	CAAAAA	0.71
6803t24	tRNA-Asn	tgcTATGAT	17	TGTGTA	1.08
6803t25	tRNA-Thr	tgcTATTCT	17	GACCCT	0.78
6803t27	tRNA-Lys	tgtTAACGT	16	TGTGGT	0.71
6803t30	tRNA-Gly	tttTACGAT	17	TAGGCG	1.04
6803t32	tRNA-Ala	tgcTAGAAT	17	TAACGA	0.90
6803t33	tRNA-Arg	tgtTACGCT	16	TCCGAA	1.50
6803t35	tRNA-Cys	tgtTATAAT	18	TTTGGT	1.96
Similar or lower expression in ΔsigBCDE than in the control strain					
slr0009	Rubisco operon	tgaTAAGAT	18	TAAGTA	0.36
slr2006	Ndh operoni	ggaTAGAAT	18	TTGCTA	-0.58
slr2008	Mrp-Ndh operoni)	tgaTAACCT	18	TGGGCA	-0.43
slr1279	ndhC operon	tccTATAAT	16	TGGTCT	-0.71
sll1732	ndhF operon	cccTATGAT	16	TGATCT	-0.31
sll0519	ndhA	gacTATACT	16	TAACCA	-0.30
slr1834	psaAB	ccgTATTAT	16	TTGCCT	0.13
slr1311	psbA2	cttTAGACT	17	TTACAA	0.00
sll0849	psbD	ttaGAGAAT	17	TTTCCA	-0.43
slr2067	apcA	tgaTAAAAT	17	GTTCGT	-0.07
sll1577	cpcB	gtaTAAAGT	17	ACATAA	0.04
slr0749	chlL	gttAATAAT	17	TCTCCA	-1.67
sll1091	terpenoid biosynthesis	tgaTAGGAT	17	ACCCCT	-0.32
sll1184	ho1 (heme oxygenase)	tagGCTAAT	18	GTACGA	-0.85
slr0040	cmpA	cttAATAAT	16	TTATCT	-0.34
ssl2084	acp	cgtTAAGAT	16	AGTGCA	0.27
slr0737	psaD	tgtTACAGT	16	GGAGCA	-1.18
slr2033	rubredoxin, HypA	tgtCATAAT	18	TGGGGA	0.30

Table S5. Primers used in this study and the length of the PCR products

Genes	Primers	Product, bp
sll3446-rpl31	5'-TGCCAAAGTCACCTGTAACG-3'<br>5'-AGGAGATCCAACTGGGAGGT-3'	756 bp
rpl31-rps9	5'-CAAGCTAACGATTCCAGCAA-3'<br>5'-TACGCATAAAGCGGTCAACA-3	653 bp
rps9-truA	5'-CTCTGGACAACGCAGTCTGA-3'<br>5'-ACCATGGGCATTAACCAAAA-3'	925 bp
truA-rpoA	5'-GTCTGAAGCGAGCCCAGAT-3'<br>5'-CACTGGGTCGTCAAAATGG-3'	837 bp
rpoA-rps11	5'-CCGATGTCCGAGTCAAAGAT-3'<br>5'-CGCCTAGAATAGTGGCGAAC-3'	472 bp
rpoA-rpl36	5'-AGCTTCCGTTAAGAAAATGTGTG-3'<br>5'-CGCCTAGAATAGTGGCGAAC-3'	1332 bp
rps13-adk	5'-TTTTTCTCGATGAGTTGTTGGTT-3'<br>5'-ACTGGTAAACCTTGGCGATG-3'	1248 bp
rps13-infA	5'-GCGATCGGGTCAAAGTAGAA-3'<br>5'-ACTGGTAAACCTTGGCGATG-3'	709 bp
adk-secY	5'-TACGCCAGTCTGATCGTCAA-3'<br>5'-TTGGTGGCAATGGCTTCTAC-3'	988 bp
secY-rpl15	5'-CACCCTAGAAAGCCTGATGG-3'<br>5’-TCCACATCACAAACATCGAG-3’	787 bp
rpl15-rps5	5'-TAAAGCTGGGGACGTAATCG-3'<br>5'-GCATGGACGGTGAGAGCTAC-3'	797 bp
rps5-rpl18	5'-CGCTCCAATGACCACATCTA-3'<br>5'-GGCAATTACCCCAGTACCAG-3'	648 bp
rpl18-rpl6	5'-AAGAAGGGGAAACCATCACC-3'<br>5'-GCTTTTACCCGTCCGTGATA-3'	733 bp
rps8-rpl6	5’-CATTTCCACCGGCTTACTGT-3’<br>5’-CACAATTTCCGACATGCTCA-3’	792 bp
rps8-rpl5	5'-CCAAGGCTTTGGAGTCTTCC-3'<br>5'-GCAAACCAGGCTTACTGACC-3'	751 bp
rpl5-rpl24	5'-AGGTGGTGGTCAAAGGAGTG-3'<br>5'-TCAAAGCTGTTGGGGCTAAT-3'	695 bp
rpl24-rps17	5'-TGGCGATTAAAGAACGTGTG-3'<br>5'-CGATGATTTCGCCTGTTTTT-3'	980 bp
rps17-rpl16	5'-CACCCTCAACTTCGGTGATT-3'<br>5'-TGATTACTTGCCAGCGTTTG-3'	800 bp
rpl16-rps3	5'-CCGTCAGATTCGAGTCAATG-3'<br>5'-TCCAGCTAGTTCAAACATGACG-3'	794 bp
rps3-rpl22	5'-AAATTCGGGGTCGTTCCTAC-3'<br>5'-AAAATGCCGTAGGTGGTCAG-3'	900 bp
rpl22-rpl2	5’-AGCGTCACTTAGGTCGTCGT-3’<br>5’-GCAAAGGCTTGGCTAACAAC-3’	774 bp
rpl2-rpl4	5'-CAAACCAGAAAAACGGGTGT-3'<br>5'-GCCGGAATATTTTGCTTGTT-3'	764 bp

rpl4-rpl3	5'-GTAACATGACCCACGGTTCC-3'<br>5'-TCACCGTAAACCTCCTGGAT-3'	916 bp
rpl3-mrgA	5'-ATTTGAGCACATCCCGACA-3'<br>5'-GTTTTCGCAGGGGTGATGT-3'	1487 bp
sll1792-rpoC2	5'-GGATTGATTGAAACCCAGGA-3'<br>5'-AGTTCAACCAACCGAACGTC-3'	1172 bp
sll1791-rpoC2	5'-GGATTGATTGAAACCCAGGA-3'<br>5’-GGGTAACATTGCCCATTGAC-3’	653 bp
rpoC2-rpoB	5'-GATGACATGCAGGGTCGTAA-3'<br>5'-CCGAAGTACCGTTCCAGGTA-3'	687 bp
rpoB-tatD	5'-AGCCTTTGTCCGCCATGT-3'<br>5'-CCGATGAAGACTTCCTGCTC-3'	502 bp
tatD-rps20	5'-AATATCAAGTCCGCCCTGAA-3'<br>5'-TAAAAAGGTTGCGGGCATTA-3'	1189bp
rps20-hik3_1	5’-GGAGAAATTGTCGCCTTTGA-3<br>5'-TGGTGTTGATCGTGTCGAGT-3'	618 bp
rps20-hik3_2	5'-ATTGAAAGAGAGCGGCAAAA-3'<br>5'-TGGTGTTGATCGTGTCGAGT-3'	1371 bp
sll1180-rpoC1	5'-CCCTAAGGGTCTCCACTTCC-3'<br>5'-TTGGTCACTTCCCCCACTAC-3'	348 bp
rpoC1-sll1178	5'-GAGGTCATCAAAACCGAGGA-3'<br>5'-TATCTGAAAGGTCCCGCT -3'	951 bp
sll1533-slr1634	5'-GGGAGCTGGCATAAACTGAG-3'<br>5'-CTGTCCCTCCCAAAAGACTG-3'	945 bp
slr1634-sll1532	5'-TCGTCAAGGGAAGGAGTAGG-3'<br>5’-TTTCTCGCCGAACTTTATCG-3’	684 bp
slr1634-rpoZ	5'-TCGTCAAGGGAAGGAGTAGG-3'<br>5'-TCCTAGGAGCCGCATCAAAC-3'	1350 bp
sll1532-rpoZ	5’-TCCTAGGAGCCGCATCAAAC-3’<br>5’-GCTGGGAAAGTCCAGGTTGA-3’	795 bp
rpoZ-slr1636	5'-ATCTTCGGAGCGATTTTCCT-3'<br>5'-TCCATGGTTAATGGATGCAC-3'	323 bp
rpoA probe	5’-GGAATCCAGCACCAGAAAAA-3’<br>5’-TTTCGTGGGGCAGGGTAATA-3’	905 bp

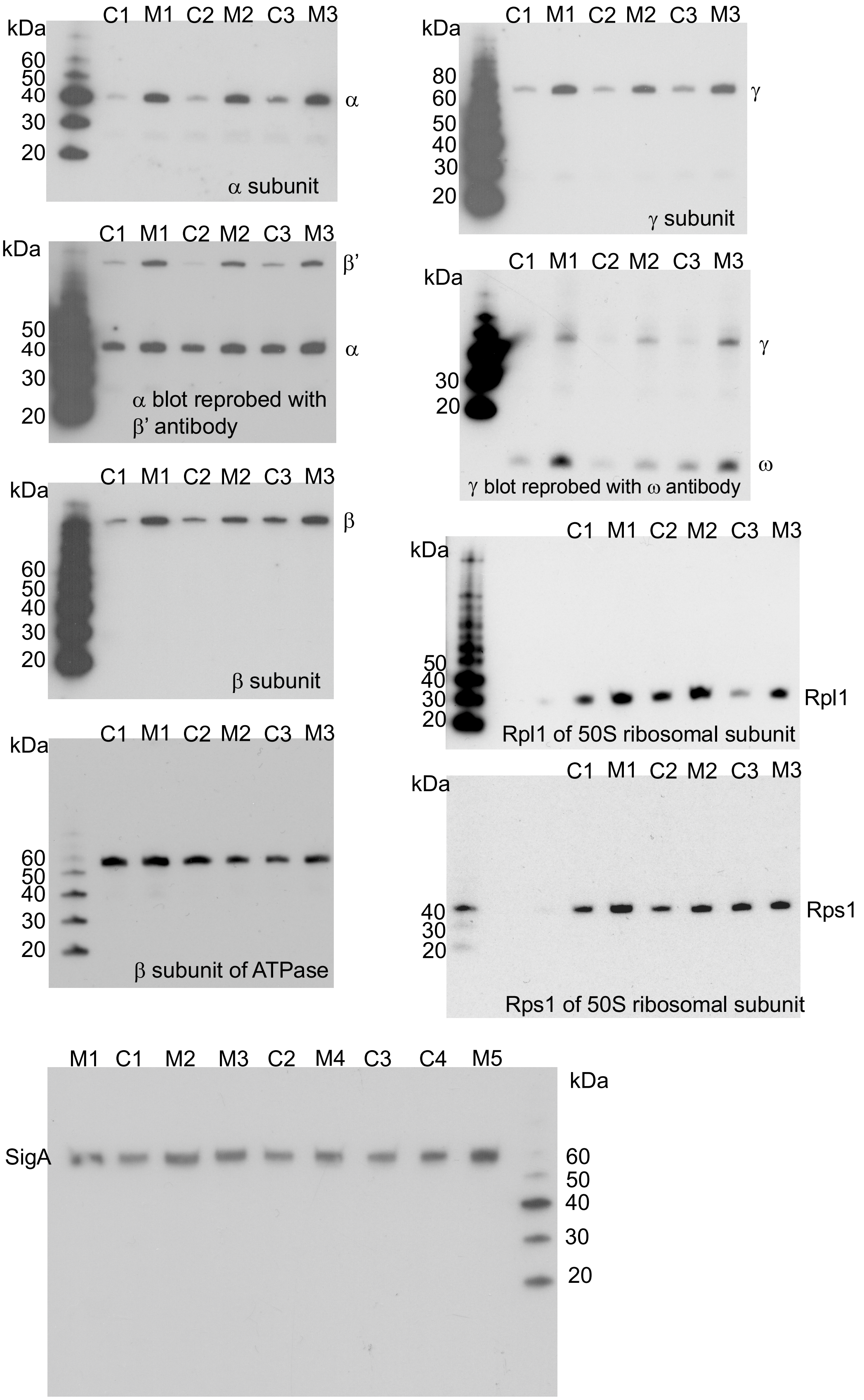

Supplementary Figure S1. Original western blots for Figures 1 and 2. The proteins isolated from independent biological replicates of the control (C1-C4) and Δ*sigBCDE* (M1-M5) strains were separated with SDS-PAGE, and the RNAP core proteins or SigA factor were detected with specific antibodies as indicated in each blot.

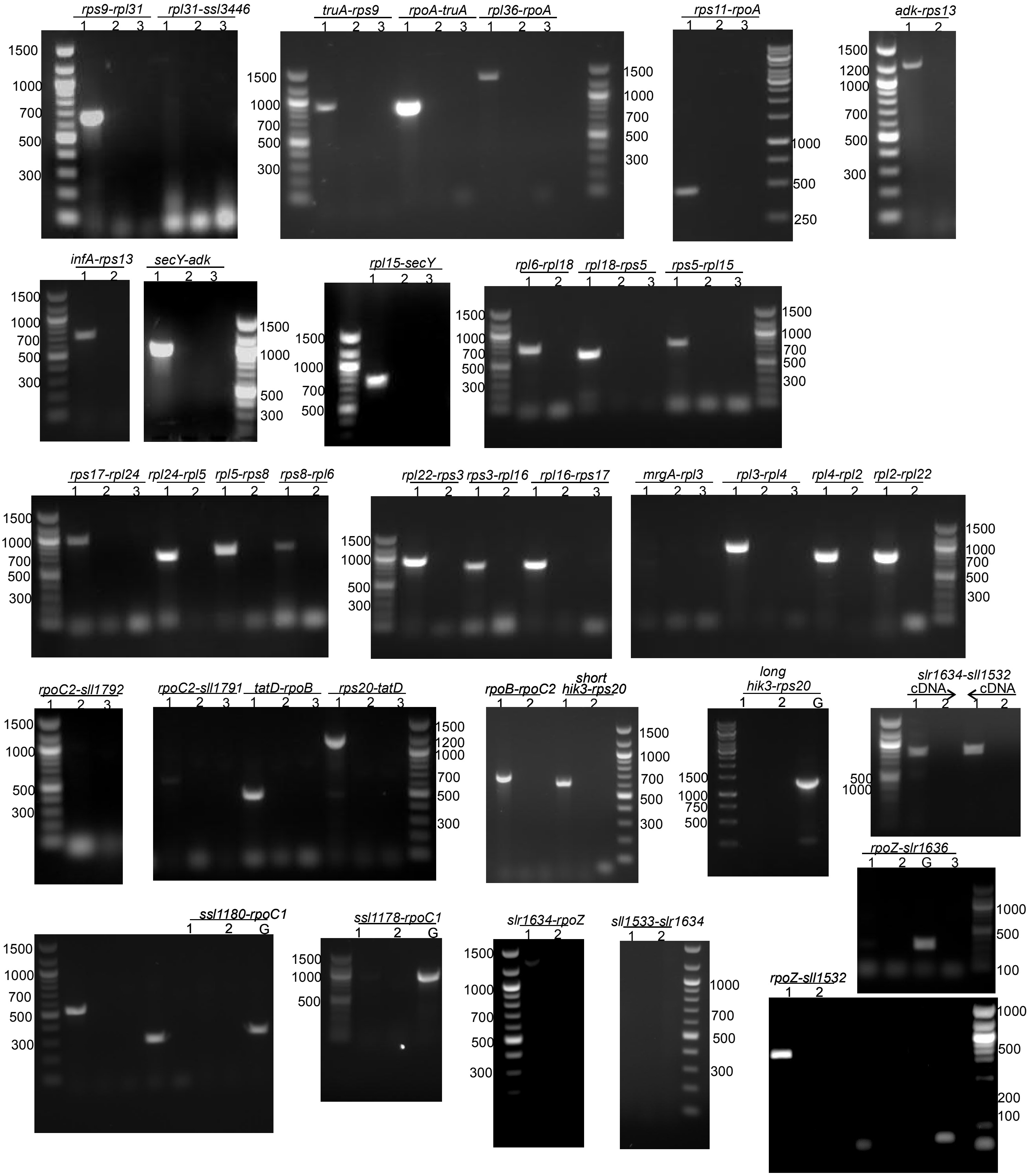

Supplementary Figure S2. Original PCR gels for operon analysis. DNA-free RNA was isolated and cDNA synthesis was performed using random hexanucleotide primers. Specific primer pairs, as indicated for each sample (see Table S5 for the primer sequences), were then used in subsequent PCR reactions. Reactions with reverse transcriptase (1), control reactions without reverse transcriptase (2), control reactions with genomic DNA (G) and PCR reactions without added template (3) are indicated. Unmarked wells contain samples not related to this study.

GTCGTGGTCAGACCACAATTAAGCCCCATTTTTTTGTTATCGAGGCTACAATCAATGAAGTCTTTTTTCACTGATTCATCAAG
CTCTACCGCTGACTAGCCTTCTCTAACACTTTTACGCCTGAATCCTTGCATTCTGTTTTTCCCCAACGAAGAAGTTAGAGAAC
CCCGAATCAATAGACTGACCGAGCGCACATCATCTAGCAACGCCTAGGGGTGGCCTCAACTTTGACTGACCAGCTAATTTT
GTACACGACTTAGGAGTTAACGAGGAAAACGGCATGACCCAGACGAAAGAGCC

Supplementary Figure S3. The upstream region of the *sigA* gene. Initiation codon of the *sigA* gene is shown with a bold italic font. Orange font shows transcription initiation site predicted by Kopf et al. (2014) DNA Res. 21:527-539 and possible -10 and -35 regions are indicated in red. Two possible promoters predicted by Imamura et al. (2003) J Mol Biol 325:857-872 are shown in green and in light green.

AGTTACCGGTGTCGAGCGAAGTTTTTTTCTATGGTTGAAGCCGTTACTATGACTGTGATTCAGGGATTTTGAAAAATATTTTGTGAA
AAGGTGTTGACTTTTCTTGCGGTATTCCGTATATTGATAAATG

Supplementary Figure S4. The identical promoter regions of the two *rrn* operons of *Synechocystis* sp. PCC 6803. The first nucleotide transcribed is blue and -10 and -35 elements red.

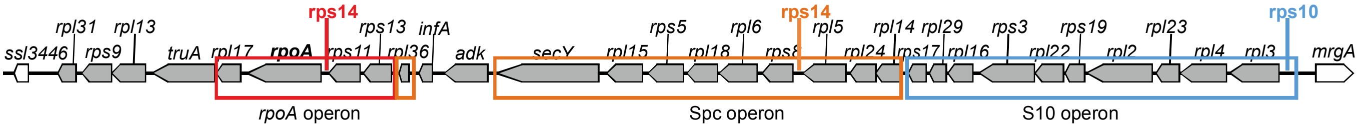

Supplementary Figure S5. *Synechocystis rpoA* opeon (grey arrows) and *E.coli rpoA* (red), Spc (orange) and S10 (blue) operons.
